# Clinician Review of Advanced Care Planning for Older Surgical Patients Requiring Intensive Care

**DOI:** 10.1016/j.jcjq.2023.09.008

**Published:** 2023-10-11

**Authors:** Joseph A. Lin, Alexis Colley, Logan Pierce, Emily Finlayson, Rebecca L. Sudore, Elizabeth C. Wick

**Affiliations:** Department of Surgery, University of California, San Francisco (UCSF) School of Medicine.; Palliative Medicine, UCSF School of Medicine.; Department of Medicine, UCSF School of Medicine.; Department of Surgery, UCSF School of Medicine.; Department of Medicine, UCSF School of Medicine.; Department of Surgery, UCSF School of Medicine.

## TO THE EDITOR

Advance care planning (ACP) reduces decisional conflict, depression, and post-traumatic stress disorder in patients and caregivers when making serious medical decisions. ^1–5^ Reappraisal, discussion, or review of previous ACP documentation is imperative at key junctures in patients’ health trajectories. For existing ACP to be helpful during the surgical episode, it must be accessible by clinicians in the electronic health record (EHR). It is unknown how clinicians caring for critically ill older surgical patients access ACP in the EHR.

## METHODS

We included all patients aged ≥65 years admitted to the ICU at a single academic center from 2018 to 2020. To determine clinician patterns of ACP review, we extracted clinical, demographic, and clinician usage data from the EHR. The primary outcome was ACP review, defined as a clinician opening the ACP section of a patient’s EHR, including the timing in relation to ICU admission. The ACP section contains relevant documentation (scanned forms and clinician-authored notes), surrogate information, and code status orders. ICU admission was chosen as a key time point representing a change in patient trajectory that should prompt review of the patient’s goals and preferences. Our main outcome was timely ACP review, defined as a clinician opening the ACP section of a patient’s EHR anytime between hospital admission and 48 hours after ICU admission. Surgical patients were defined as those admitted to a surgical service or who underwent surgery during the admission. Elective, urgent, and emergent operations were included. Multivariate logistic regression was performed. The Institutional Review Board approved this study.

## RESULTS

**Figure 1:**
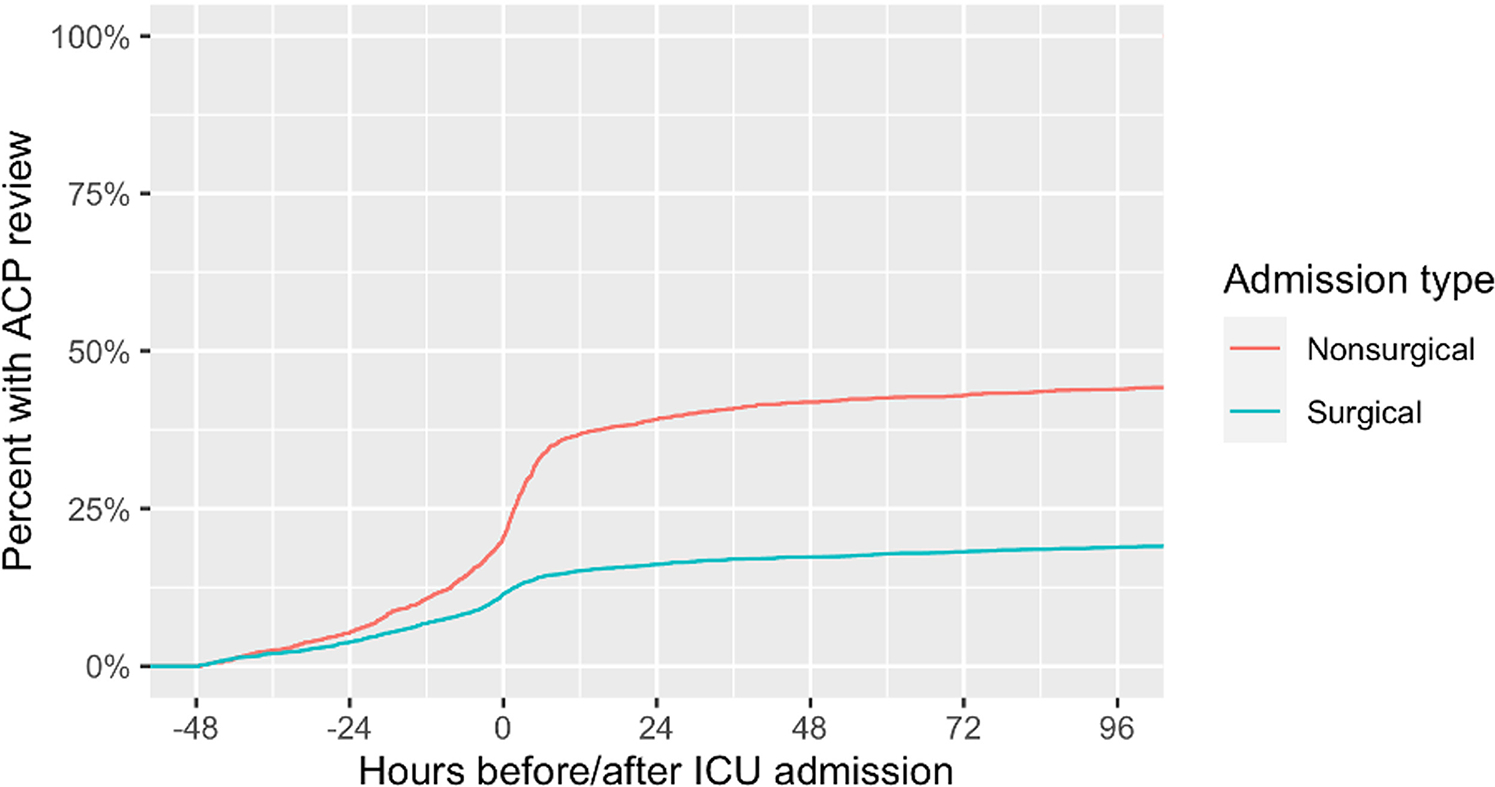
This bar graph shows the frequency of advanced care practice (ACP) review based on existing documentation.

**Figure 2:**
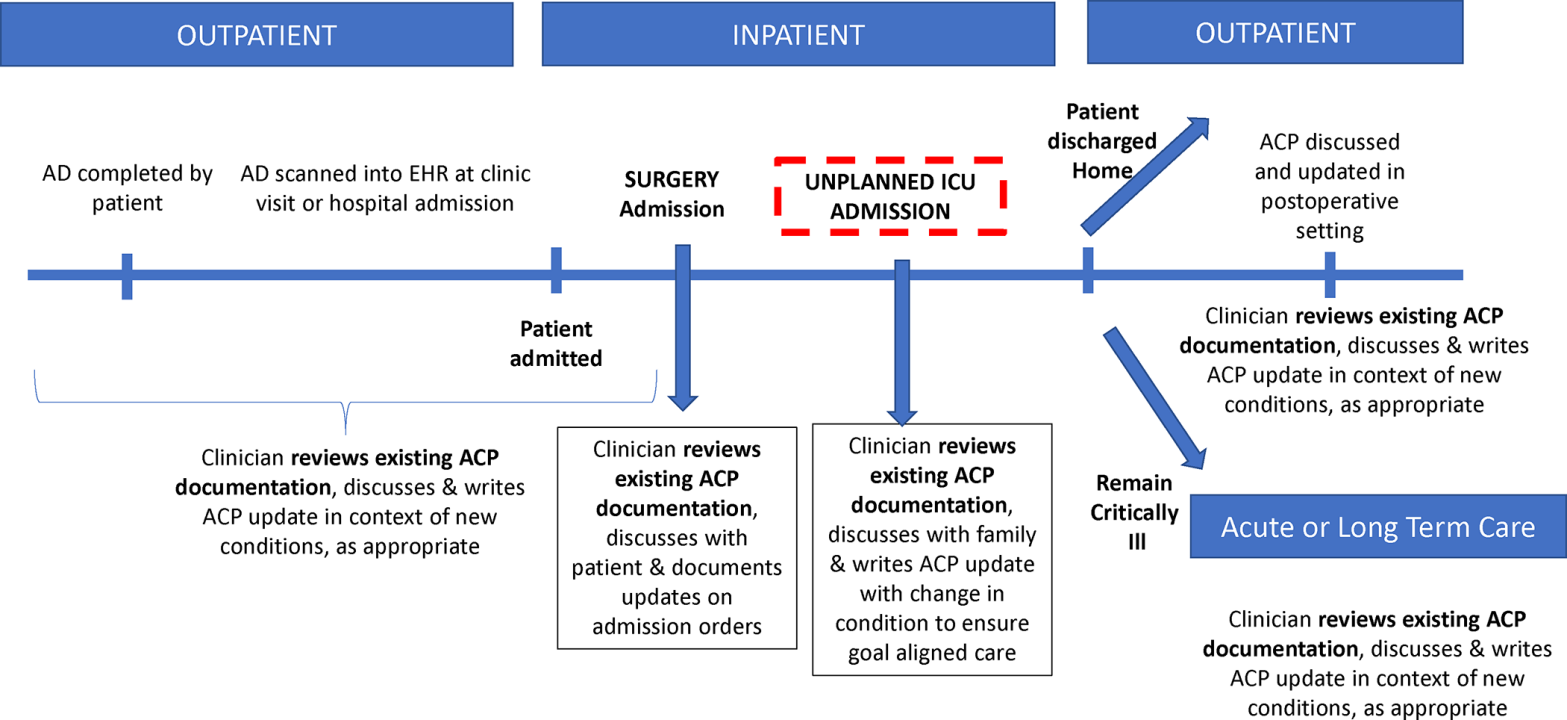
This graph shows the cumulative rate of advanced care practice (ACP) review relative to time before or after ICU admission.

Of 6,519 older patients admitted to the ICU, 4,352 (66.8%) were surgical patients, of whom 3,911 (89.9%) had a surgical admitting service, 3,217 (73.9%) had surgery, and 2,776 (63.8%) had both. Among surgical patients, 1,342 (30.8%) had ACP documentation in the EHR prior to ICU admission: 947 (21.8%) had only scanned forms, 286 (6.6%) had clinician-authored notes, and 109 (2.5%) had both. The frequency of timely ACP review for surgical patients was 21.0% (914 of 4,352), and ACP review was most likely for those with more extensive documentation (Figure 1). ACP review increased for all patients around the time of ICU admission but was higher for nonsurgical patients at all time points (Figure 2).

In regression analysis, ACP review within 48 hours of ICU admission was significantly more likely with older age (per-decade odds ratio [OR] 1.51, 95% confidence interval [CI] 1.36–1.67, *p* < 0.001) and Chinese (Mandarin or Cantonese) primary language compared to English (OR 1.51, 95% CI 1.07–2.12, *p* = 0.02), but less likely for partnered patients compared to single (OR 0.82, 95% CI 0.70–0.96, *p* = 0.02) and for patients admitted to surgical services compared to medical services (OR 0.33, 95% CI 0.27–0.41, *p* < 0.001). Compared to nonsurgical admitting services as a reference, Gynecologic Surgery was the only surgical admitting service not associated with significantly decreased likelihood of ACP review.

Of 329 (7.6%) surgical admissions that ended in death, only 64 (19.5%) had ACP review within 48 hours of death. Mortality of the surgical cohort at one year was 22.9% (*n* = 998). Overall, 2,823 unique clinicians performed timely ACP review. These clinicians included 1,185 (42.0%) registered nurses, 765 (27.1%) attending physicians, 271 (9.6%) residents, 121 (4.3%) advanced practice providers, and 481 (17.0%) other providers.

## DISCUSSION

Even when ACP documentation was readily accessible in the EHR, ACP review was inconsistent for surgical patients despite critical illness and significant mortality. Meaningful ACP requires not just discussion and documentation but also review of patients’ goals and preferences in the event of significant change in condition across the care continuum (Supplemental Figure 1). Notably, Gynecologic Surgery and nonsurgical admission services outperformed surgical services in terms of timely ACP review when patients were admitted to the ICU, suggesting that the challenges are related to specialty-specific practice patterns. Future work should focus not just on completing ACP but also promoting ACP workflows across the continuum of care to ensure that patient goals and preferences are reviewed, updated, and followed.

## Supplementary Material

Slideshow
