# Supplementary material for: Clinician Review of Advanced Care Planning for Older Surgical Patients Requiring Intensive Care
Source: Jt Comm J Qual Patient Saf. Author manuscript; Available in PMC 2024 Feb 23. (PMC10890795; doi:10.1016/j.jcjq.2023.09.008)
Supplement: Slideshow [file NIHMS1962114-supplement-Slideshow.pptx]

## Slide 1
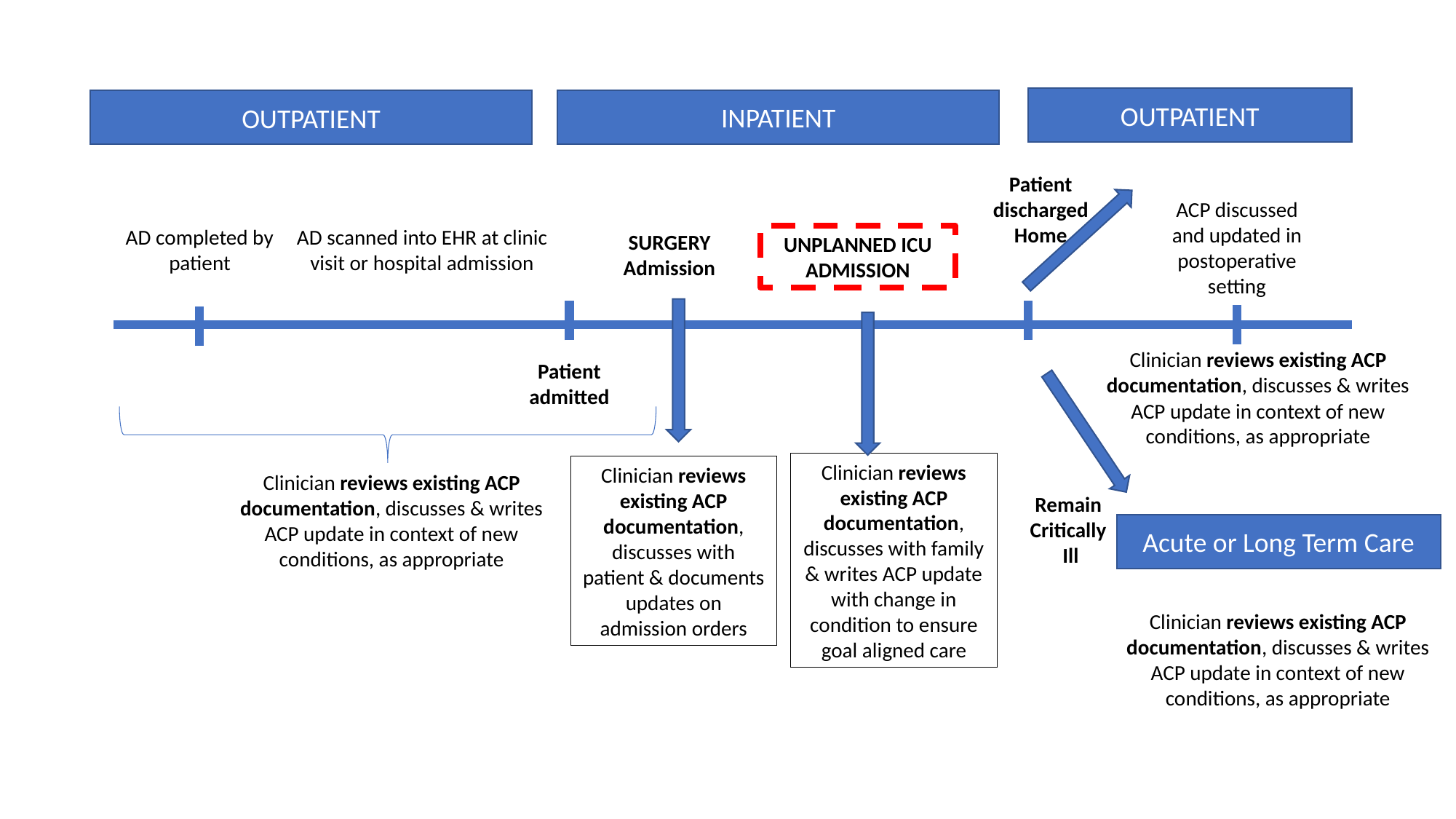

OUTPATIENT
INPATIENT
OUTPATIENT
Patient discharged
Home
ACP discussed and updated in postoperative setting
AD completed by patient
AD scanned into EHR at clinic visit or hospital admission
SURGERY
Admission
UNPLANNED ICU ADMISSION
Clinician reviews existing ACP documentation, discusses & writes ACP update in context of new conditions, as appropriate
Patient admitted
Clinician reviews existing ACP documentation, discusses with family & writes ACP update with change in condition to ensure goal aligned care
Clinician reviews existing ACP documentation, discusses with patient & documents updates on admission orders
Clinician reviews existing ACP documentation, discusses & writes ACP update in context of new conditions, as appropriate
Remain Critically
 Ill
Acute or Long Term Care
Clinician reviews existing ACP documentation, discusses & writes ACP update in context of new conditions, as appropriate
